# Letter from a Country Doctor

**Published:** 1887-07

**Authors:** E. W. Lane

**Affiliations:** Scarboro, Ga.


### LETTER FROM A COUNTRY DOCTOR.

*Editors Atlanta Medical and Surgical Journal:*

I am proud to see the article in THE ATLANTA MEDICAL AND SURGICAL JOURNAL, from Dr. Todd, on poke-root, ergot and mistletoe, as read before the Atlanta Society of Medicine. I thank him for allowing it published, that others may profit by his experience and observation. What he says of poke-root tallies with what I have found to be true. I have had many years' experience in the use of the drug and it seldom failed me; I remember of no instance when it failed me where its use had been commenced before pus had formed. I look upon poke-root as being as near a specific in such cases as drugs possibly can be in any disease. We make our own tinctures, as we find them to be equally if not more efficacious than those we buy of the drug-houses.

What Dr. Todd says of mistletoe is equally true, so far as my experience goes, and it is a remedy much used in this section by the country doctor. Our experience reaches further than as given in your article. We find it to be a sure and safe remedy. Its action seems to be directed more on the fundus of the uterus than to the whole organ. Ergot is not a safe remedy, as its action takes in the whole organ. Mistletoe is the remedy to expel a retained placenta—it has never failed us. Ergot is the remedy not to give in such cases.

Mistletoe was the subject of discussion at our last meeting of the Ogeechee Medical Association. It was brought out that Dr. T. E. Johnson had successfully treated a case of hydatids of the uterus—the remedy expelling the tumors in a few days. It is used also to expel a dead fœtus.

What Dr. Todd says of wild yam is new to me. I would like to know the plant. The wild potato grows abundantly in this neighborhood, but the description given in the books of wild yam don't suit it. Whenever the plant grows in this locality, I prefer to use it, but if I am not able to find it here, I will get it of some reliable drug-house and try it in the next suitable case.

What he says of infantile colic, so far as its nature and treatment are concerned, is also new, but as I have had eight children in my own family, and have been actively engaged in the practice of medicine for near the third of a century, it could hardly be supposed that I would have been so fortunately situated as to escape, but while I have seen it in all its glory and been bedeviled by it at home and abroad, I have not as yet been able to cure a case, but the next one I am called to I shall give his prescription a fair trial.

I have never used ergot for hemorrhoids in the way Dr. Todd says, but will, at the first opportunity, give it a trial. I am glad that ergot is good for something, as I was about to give it up. A few years ago it had a big run. It was recommended for almost every disease that the human body was heir to, but at this time it is seldom used for anything, it having been found that other remedies better fill the place that it formerly occupied.

Fraternally yours, E. W. LANE, M. D.

*Scarboro, Ga., June 9, 1887.*

